# Fine tuning of the side-to-side tenorrhaphy: A biomechanical study assessing different side-to-side suture techniques in a porcine tendon model

**DOI:** 10.1371/journal.pone.0257038

**Published:** 2021-10-05

**Authors:** Christina J. Wilhelm, Marc A. Englbrecht, Rainer Burgkart, Carina Micheler, Jan Lang, Christine S. Hagen, Riccardo E. Giunta, Nikolaus Wachtel

**Affiliations:** 1 Division of Hand, Plastic and Aesthetic Surgery, University Hospital, LMU Munich, Munich, Germany; 2 Department of Orthopaedics and Sports Orthopaedics, Klinikum Rechts der Isar, School of Medicine, Technical University of Munich, Munich, Germany; 3 Department of Mechanical Engineering, Institute for Machine Tools and Industrial Management, Technical University of Munich, Munich, Germany; 4 Department of Mechanical Engineering, Chair of Non-Destructive Testing, Technical University of Munich, Munich, Germany; Universidade Federal Fluminense, BRAZIL

## Abstract

Recent studies conclude that a new technique for tendon transfers, the side-to-side tenorrhaphy by Fridén (FR) provides higher biomechanical stability than the established standard first described by Pulvertaft (PT). The aim of this study was to optimize side-to-side tenorrhaphies. We compared PT and FR tenorrhaphies as well as a potential improvement, termed Woven-Fridén tenorrhaphy (WF), with regard to biomechanical stability. Our results demonstrate superior biomechanical stability and lower bulk of FR and, in particular, WF over PT tenorrhaphies. The WF and FR technnique therefore seem to be a notable alternative to the established standard tenorrhaphy as they display lower bulk and higher stability, permitting successful immediate active mobilization after surgery.

## Introduction

Tendon transfers with a subsequent side-to-side tenorrhaphy are predominantly used to restore limb-function after trauma of the central or peripheral nervous system and/or trauma impairing muscle (tendon) function of the extremities [1, 2]. This surgical technique is commonly performed in foot and ankle as well as in hand and plastic surgery. Common examples include the treatment of patients with common peroneal nerve palsy (foot drop) and insertional or noninsertional tendinopathy of the Achilles tendon [3, 4]. Moreover, side-to-side tenorrhaphies are used to restore critical grasping motions of the hand after brachial plexus palsy [5, 6].

Here, an optimal suture stability is essential, allowing for early mobilization and, thus, reduced adhesion formation and post-operative recovery time [7, 8]. The established standard technique for a tendon transfer was first described by Pulvertaft (PT) *et al*. [9–11]. Interestingly, recent studies demonstrated that a new technique, the side-to-side tenorrhaphy by Fridén (FR) *et al*., provides both efficient force transmission and high suture strength. Indeed, repair stiffness, load at first failure and ultimate failure load of the FR suture were significantly higher when compared to PT sutures [7, 8, 12, 13].

The aim of this biomechanical study was therefore to optimize the technique of side-to-side tenorrhaphies. We assessed the biomechanical properties of PT, FR, and a potential improvement of the FR technique, termed Woven-Fridén (WF) tenorrhaphy.

## Materials and methods

### Ethics statement

Ethical approval for this study was obtained from the Ethics Committee of the Medical Faculty at LMU Munich, Germany (Approval Number: 19–142). Tendons were obtained from porcine hind limbs. These were purchased from a local butcher. The Ethics Committee for Animal Experiments, LMU Munich, granted a formal waiver for current and future experiments using tendons from porcine hind limbs.

### Inclusion, exclusion and randomization

The extensor tendons of porcine hind limbs were harvested as previously described by Fuchs *et al*. [14]. After harvesting, tendons were stored in a vacuum bag and fresh-frozen at -28°C. Specimens were thawed in water at 37°C directly before performing the sutures [15, 16]. Block randomization was used to determine which tendon pairs were used in experimental groups and to distribute the tendons of each limb to the three groups of one experimental series in a balanced manner (S1 Data). For each experimental group, 12 side-to-side tenorrhaphies were tested. Thus, a total of 72 sutures were tested.

### Tenorrhaphies

**Fig 1:**
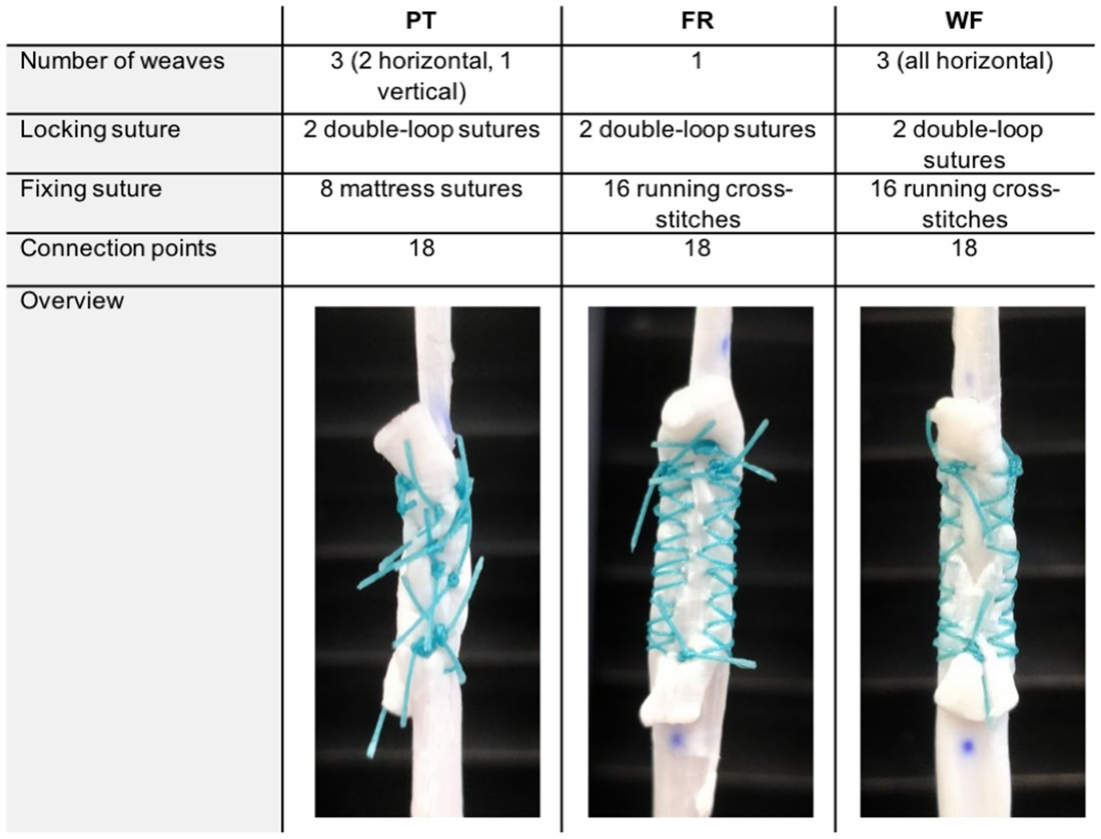
Overview of suture techniques used. The different techniques Pulvertaft (PT), Fridén (FR) and Woven-Fridén (WF) are illustrated: The donor tendon (above) was woven through incisions in the recipient tendon (below). For each suture technique the same amount of connection points between the tendons was used. The threads were cut in standardized fashion at 10 mm length.

**Fig 2:**
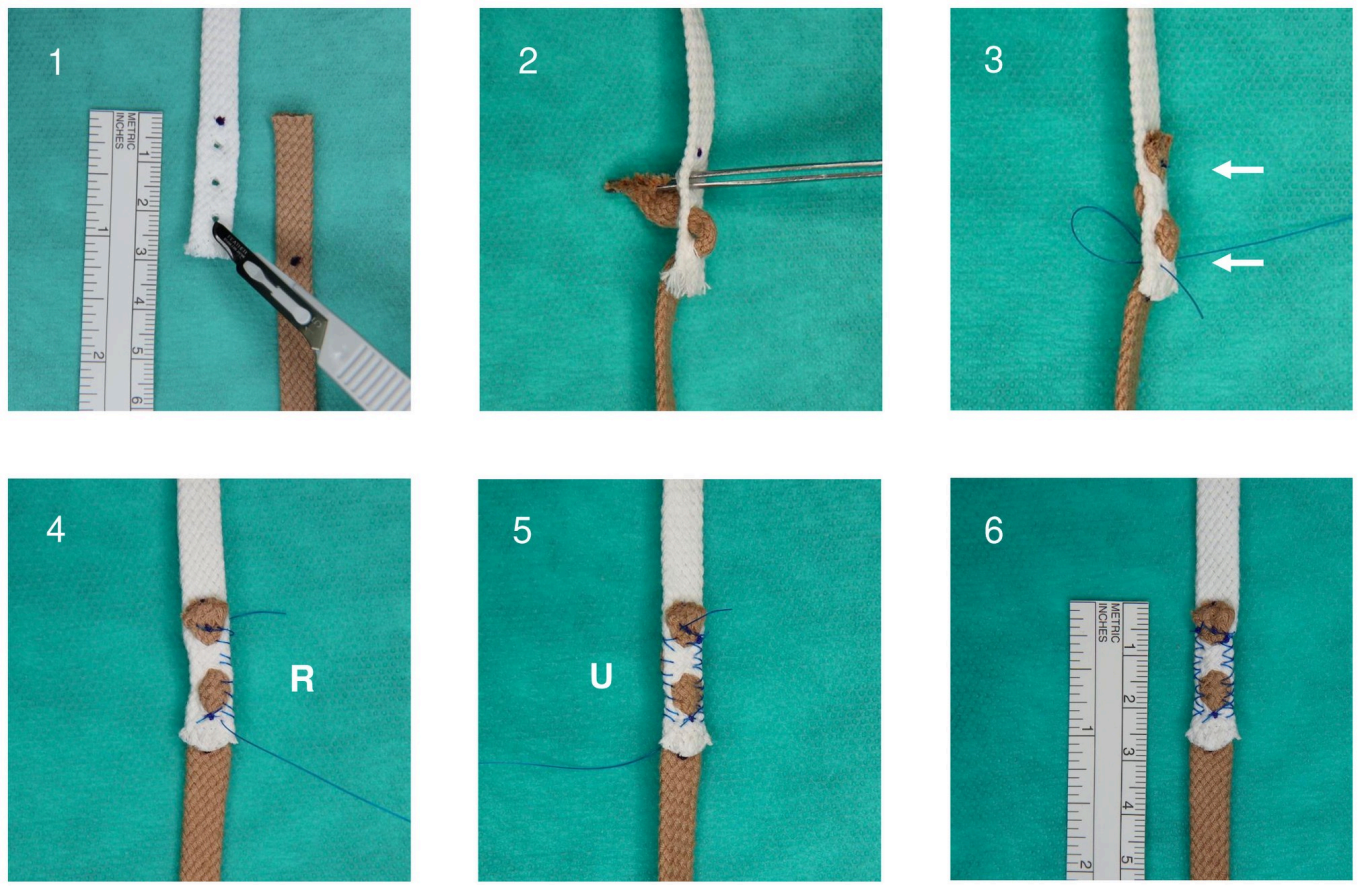
Schematic model of the Woven-Fridén tenorrhaphy (WF) (30 mm tendon-tendon overlap) using brown and white strings. **1.** Mark incisions and overlap for donor- (brown) and recipient tendon (white) with a surgical pen and make three incisions in the recipient tendon using a No. 15 scalpel blade. **2.** Interlace the donor tendon (brown) through the recipient tendon (white). **3.** Perform two double-loop sutures at proximal and distal end of tenorrhaphy (arrows). **4.** Perform eight running cross stitches at the radial side (R) and **5.** ulnar side (U) of the tenorrhaphy. **6.** WF tenorrhaphy (the overlap has been reduced to approximately 27 mm due to interlacing of tendons).

PT and FR tenorrhaphies were performed as described previously by Brown *et al*. [7] (Fig 1). Additionally, we tested a varied approach to the FR technique, termed WF (Fig 2). Each tenorrhaphy was performed with a 30 mm tendon-tendon overlap. Ethibond^™^ 3–0 (Ethicon, Inc. Somerville, NJ, USA) was used as suture material.

### Suture characteristics and biomechanical testing

Tendon and suture diameter as well as the length of overlap were measured with a digital caliper after tenorrhaphy completion. The bulk ratio of tenorrhaphies was determined by dividing the cross-sectional area of tendons [13]. For biomechanical testing, stiffness (resistance of sutures to deformation), first failure load (first local maximum of force in the load-deformation curve), and ultimate load (highest force (N) achieved before ultimate failure) were measured as described previously [7, 12, 13, 17].

All experiments were performed following a standardized protocol, similar to protocols of previous studies [7, 12, 17]. All mechanical tests were performed using a calibrated tensile testing machine (Zwicki 1120, ZwickRoell GmbH & Co. KG, Ulm, Baden-Württemberg, Germany). A preload of 2 N was applied to minimize slack and 5 preconditioning cycles with a deformation of 5% of the distance between the clamps were imposed [7, 18]. Tenorrhaphies were then preconditioned at a velocity of 10 mm/min to be elongated until failure at a velocity of 100 mm/min [7, 12, 17]. The tensile load test outputs were plotted on a load-deformation curve (TestXpert V12.0 ZwickRoell GmbH & Co. KG, Ulm, Baden-Württemberg, Germany). We used a custom written MATLAB code (MATLAB R2017b, The MathWorks, Inc. Natick, MA, USA) to determine load at first failure, ultimate load and repair stiffness. The stiffness of the tenorrhaphy was determined within the linear elastic area of the load-deformation curve. The preconditioning data were removed and by means of the coefficient of determination (R^2^), the linear elastic area was identified to calculate the stiffness of the tenorrhaphy [19]. Means and standard deviations were calculated for the different groups. Tensile testing was filmed using a Legria HF M31 video camera (Canon Co. Ltd., Ohta-ku, Tokyo, Japan) to document the mode of failure (pull-out vs. suture breakage) [20, 21].

### Statistical analysis

Data are given as means and standard deviation (SD). One-way analysis of variance (ANOVA), followed by the Tukey-Kramer test for multiple comparisons was conducted to assess effects of tenorrhaphy techniques on bulk ratio, repair stiffness, first failure load, and ultimate load. A *p*-value of < 0.05 was considered statistically significant. GraphPad Prism 6 (GraphPad Software, Inc., San Diego, CA, USA) was used as software for statistical analysis.

## Results

**Fig 3:**
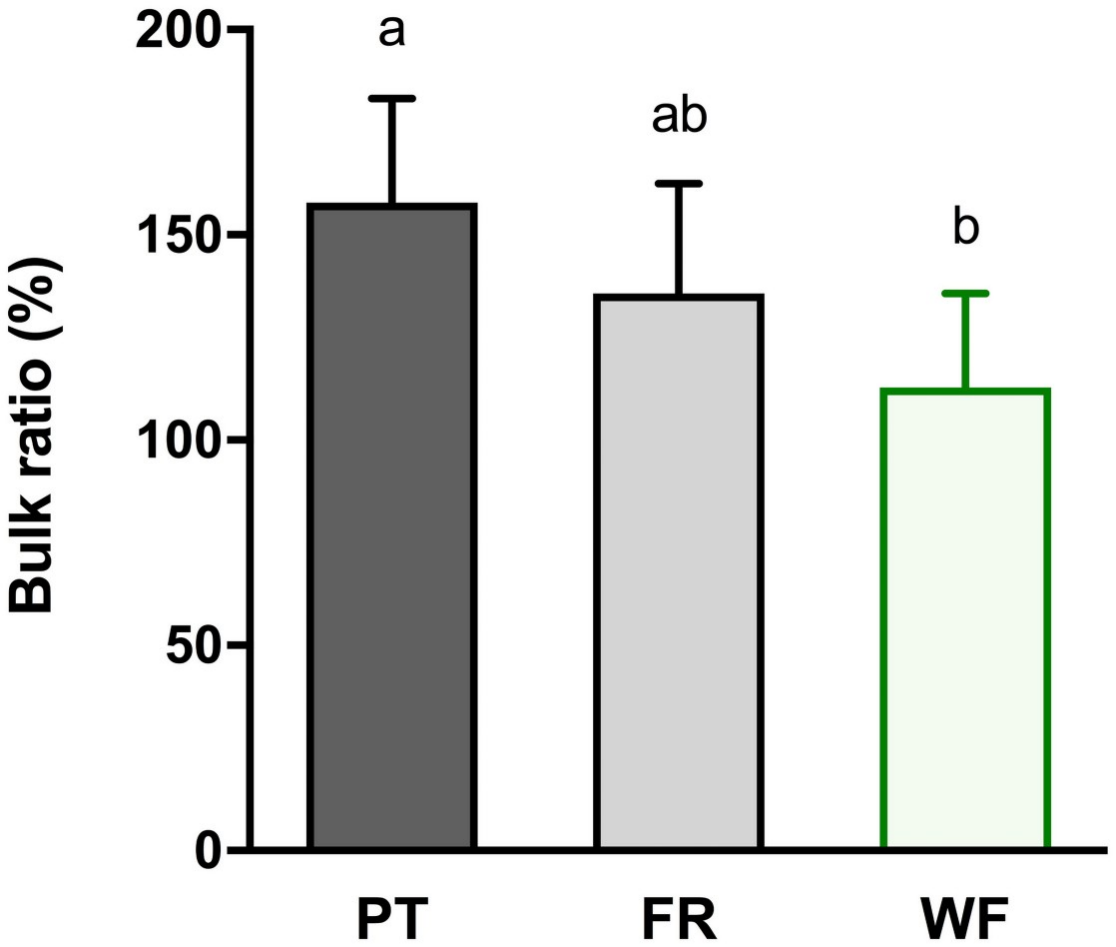
Effects of suture techniques Pulvertaft (PT), Fridén (FR) and Woven-Fridén (WF) on bulk ratio in % (ratio of the cross-sectional area of the sutured tendons and the native tendons; for details see S1 Data). Data is expressed as means, standard deviation bars are shown. Different superscripts indicate statistically significant differences among groups at *p* < 0.001. For each experimental group, 12 side-to-side tenorrhaphies were tested.

**Fig 4:**
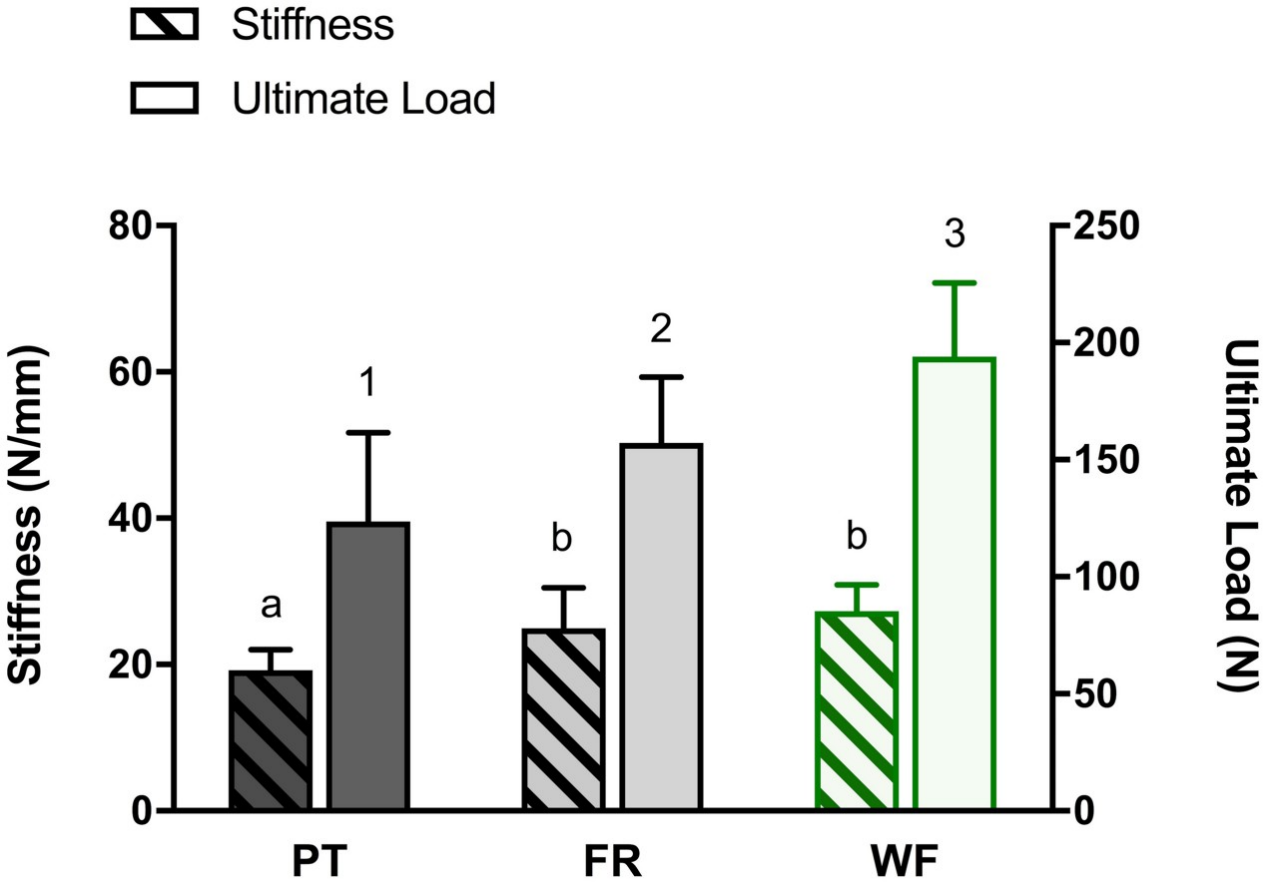
Effects of suture techniques Pulvertaft (PT), Fridén (FR) and Woven-Fridén (WF) on stiffness (resistance of sutures to deformation) in N/mm (crosshatched bars) and on ultimate load in N (single-colour bars). Data is expressed as means, standard deviation bars are shown. Different superscripts indicate statistically significant differences among groups at *p* < 0.05 and for WF vs. PT at *p* < 0.0001. For each experimental group, 12 side-to-side tenorrhaphies were tested.

When analyzing different tenorrhaphy techniques, the WF group had the lowest relative cross-sectional area (bulk ratio), which was significantly lower when compared to the PT group (*p* < 0.001) (Fig 3). Results for biomechanical analysis of different tenorrhaphies are shown in Fig 4. Ultimate load was highest in the WF group (compared to both FR and PT tenorrhaphies; *p* of 0.02 and < 0.001, respectively). Stiffness was highest for WF and FR sutures (*p* = 0.005 for PT vs. FR and *p* < 0.001 for PT vs. WF). WF sutures therefore showed highest stability while suture failure occurred earliest in the PT technique. All side-to-side tenorrhaphies failed by pull-out. For all experiments, first failure load was identical or highly similar to ultimate failure load (also see S1 Data).

## Discussion

In our study, we set out to determine the biomechanical properties of the FR side-to-side tenorrhaphy, which was recently proposed as an alternative to PT sutures with superior biomechanical stability [7, 12, 13]. Furthermore, we aimed to improve the FR technique by adding two horizontal weaves (Fig 2). We termed this variation WF.

A high bulk of tendon-to-tendon sutures can lead to friction between tendons and adjacent tissue. This results in formation of adhesions that compromise the natural gliding mechanics of tendons [18, 22–24]. Moreover, a strong mechanical tenorrhaphy is essential for an optimal clinical outcome as it permits immediate active mobilization thereby minimizing adhesion formation and providing optimal conditions for healing and mobility [8, 25]. Recent studies with a similar set-up demonstrated that the ultimate load of the FR technique was significantly higher when compared to PT sutures [7, 12, 13]. We were able to confirm these findings (Fig 4). Moreover, the highest ultimate failure load and lowest bulk formation was measured for the WF group (Figs 3 and 4). The findings of this study therefore advocate an advantage of FR and, in particular, WF side-to-side tenorrhaphies over the technique described by PT with regard to biomechanical stability as well as bulk.

A probable explanation for the higher stiffness and load bearing capacities of both FR and WF tenorrhaphies is the usage of running cross-stitches for both techniques. An advantage of these stitches seems likely as all three suture techniques had the same number of connection points (Fig 1). Indeed, a previous biomechanical study demonstrated the superior ultimate load bearing capacities of cross-stitches over mattress sutures [26]. Equally, Brown *et al*. argue that running cross-stitches permit force distribution over a larger area when compared to mattress sutures used in the PT technique [7]. The mattress suture in the PT tenorrhaphy that is tightest might act as a focused transmission of tensile load from one tendon to the next and, thus, could facilitate suture failure. Differences between WF and FR sutures may be explained by a similar mechanism: more than one weave results in a more balanced load of force in combination with a stabilizing interlocking effect between the two tendons [4]. Hereby increasing the ultimate load of WF tenorrhaphies. However, if these hypotheses prove to be correct, it has to be argued whether the usage of cross-stitches instead of mattress sutures improves the biomechanical properties of the PT suture. Thus, matching those of FR or even WF tenorrhaphies.

The conclusions of this study are predominantly limited by its *in vitro* set-up. Porcine extensor tendons differ from human tendons in size and structure and our findings on load bearing capacity may therefore differ in human tendons, in particular when tendons with a different caliber are used [14, 27–31]. Moreover, by using a cadaver model we were unable to reproduce normal tissue biology. During the healing process with a subsequent inflammatory stage, the stability of the sutured tendons is reported to decline [32, 33]. Indeed, several studies that assessed the stability of tendon sutures *in vivo* during the healing period indicate that tensile strength of sutured tendons decreases during the first weeks postoperatively [34, 35]. High suture stability *in vitro* may therefore not ensure equal biomechanical properties *in vivo*. Being aware of this limitation, we advocate for subsequent studies that assess the three sutures in an *in vivo* set-up similar to previous publications that explored tendon biology [36–38].

This study demonstrated the superior biomechanical properties of FR side-to-side tenorrhaphies over PT sutures. Additionally, our proposed modifications of the FR technique, termed WF tenorrhaphy, further improved load bearing capacities. FR and, in particular, WF tenorrhaphies therefore seem to be a superior alternative to the established standard technique for side-to-side tenorrhaphies, thereby increasing probability of successful immediate active mobilization after surgery.

## Supporting information

S1 Table Standardized experimental protocol using block randomization. A-C stand for Pulvertaft (PT), Fridén (FR) and Woven-Fridén (WF) suture techniques. Four tendons of porcine hind limbs were used: M. extensor digitalis lateralis (I), M. extensor digiti III et IV (II) M. extensor digiti III (III) and M. extensor digiti I longus (IV). Tendons were cut in half before combining the proximal part (prox.) of one tendon with the distal part (dist.) of another tendon for a suture. The lateral (I) and the medial tendon (IV) had a smaller caliber and were therefor used as donors, median tendons (II and III) were used as recipients. Every combination of donor- and recipient-tendon was equally often used for each experimental group.
(DOCX)

S2 Table Comparison of different characteristics of three different suture techniques: Pulvertaft (PT), Fridén (FR) and Woven-Fridén (WF). Values are expressed as mean (SD). Different superscripts indicate statistically significant differences among groups at *p* of at least < 0.05.
(DOCX)

S1 File Calculation of the Bulk Ratio (BR). The formula to calculate the BR is given and derived.
(DOCX)

S1 Data Tabular listing and graphical representation of all collected data. (PDF)
