# Supplementary material for: Fine tuning of the side-to-side tenorrhaphy: A biomechanical study assessing different side-to-side suture techniques in a porcine tendon model
Source: PLoS One. 2021 Oct 5;16(10):e0257038. doi: 10.1371/journal.pone.0257038 (PMC8491917; doi:10.1371/journal.pone.0257038)
Supplement: S1 Table — A-C stand for Pulvertaft (PT), Fridén (FR) and Woven-Fridén (WF) suture techniques. Four tendons of porcine hind limbs were used: M. extensor digitalis lateralis (I), M. extensor digiti III et IV (II) M. extensor digiti III (III) and M. extensor digiti I longus (IV). Tendons were cut in half before combining the proximal part (prox.) of one tendon with the distal part (dist.) of another tendon for a suture. The lateral (I) and the medial tendon (IV) had a smaller caliber and were therefor used as donors, median tendons (II and III) were used as recipients. Every combination of donor- and recipient-tendon was equally often used for each experimental group. (DOCX) [file pone.0257038.s001.docx]

**S1 Table. Standardized experimental protocol using block randomization**.

| Sample | Experimental Group | Limb | Donor | Recipient |
| --- | --- | --- | --- | --- |
| 1 | A | Limb 1 | I prox. | II dist. |
| 2 | B |  | I dist. | II prox. |
| 3 | C |  | IV prox. | III dist. |
| 4 | A |  | IV dist. | II. prox. |
| 5 | B | Limb 2 | I prox. | II dist. |
| 6 | C |  | I dist. | II prox. |
| 7 | A |  | IV prox. | III dist. |
| 8 | B |  | IV dist. | III prox. |
| 9 | C | Limb 3 | I prox. | II dist. |
| 10 | A |  | I dist. | II prox. |
| 11 | B |  | IV prox. | III dist. |
| 12 | C |  | IV dist. | III prox. |
