# Supplementary material for: Fine tuning of the side-to-side tenorrhaphy: A biomechanical study assessing different side-to-side suture techniques in a porcine tendon model
Source: PLoS One. 2021 Oct 5;16(10):e0257038. doi: 10.1371/journal.pone.0257038 (PMC8491917; doi:10.1371/journal.pone.0257038)
Supplement: S2 Table — Values are expressed as mean (SD). Different superscripts indicate statistically significant differences among groups at p of at least < 0.05. (DOCX) [file pone.0257038.s002.docx]

**S2 Table. Comparison of different characteristics of three different suture techniques: Pulvertaft (PT), Fridén (FR) and Woven-Fridén (WF).**

|  | PT  (n=12) | FR  (n=12) | WF  (n=12) |
| --- | --- | --- | --- |
| Length of overlap (mm) | 24.2 (1.2) ^a^ | 26.5 (1.0) ^b^ | 26.1 (1.1) ^b^ |
| Cross-sectional area of the native tendons (mm^2^) | 11.4 (2.0) | 10.9 (3.1) | 12.1 (2.1) |
| Cross-sectional area of the sutured tendons (mm^2^) | 17.9 (3.7) ^a^ | 14.4 (3.7) ^b^ | 13.4 (2.6) ^b^ |
| Bulk ratio (%) | 158 (25) ^a^ | 136 (27) ^ab^ | 113 (23) ^b^ |
| Repair stiffness (N/mm) | 19.2 (2.8) ^a^ | 25.0 (5.5) ^b^ | 27.3 (3.6) ^b^ |
| First failure load (N) | 121.4 (37.9) ^a^ | 154.6 (29.2) ^b^ | 194.1 (31.5) ^c^ |
| Ultimate load (N) | 123.7 (37.9) ^a^ | 157.2 (28.2) ^b^ | 194.1 (31.5) ^c^ |
