# Supplementary material for: Fine tuning of the side-to-side tenorrhaphy: A biomechanical study assessing different side-to-side suture techniques in a porcine tendon model
Source: PLoS One. 2021 Oct 5;16(10):e0257038. doi: 10.1371/journal.pone.0257038 (PMC8491917; doi:10.1371/journal.pone.0257038)
Supplement: S1 File — The formula to calculate the BR is given and derived. (DOCX) [file pone.0257038.s003.docx]

**S1 File**

**Calculation of the Bulk Ratio (BR)**

To calculate the bulk ratio $BR$, the cross-section of the sutured tendons $A_{tenoraphy}$ is divided by the cross-section of the native tendons $A_{native}$. The latter is composed of the two cross sections of donor and recipient tendon $A_{donor}$ and $A_{recipient}$.

$$BR= \frac{A_{tenoraphy}}{A_{native}} = \frac{A_{tenoraphy}}{A_{donor}+ A_{recipient}}$$

The cross-section of the tenorraphy can be described as elliptical. Accordingly, the largest and smallest diameters of the ellipse $d_{a}$ and $d_{b}$ are measured. The cross-section of the native tendons is approximately circular. To calculate the circular shape, the diameters of the donor and recipient tendons $d_{c}$ and $d_{d}$ are measured.

$$BR= \frac{\frac{d_{a}}{2} \times\frac{d_{b}}{2} \times\pi}{\left( \frac{{d_{c}}^{2}}{4} \times\pi\right)+\left( \frac{{d_{d}}^{2}}{4} \times\pi\right)}$$
