# Supplementary material for: Fine tuning of the side-to-side tenorrhaphy: A biomechanical study assessing different side-to-side suture techniques in a porcine tendon model
Source: PLoS One. 2021 Oct 5;16(10):e0257038. doi: 10.1371/journal.pone.0257038 (PMC8491917; doi:10.1371/journal.pone.0257038)

Comparison of suture techniques (PT, FR, WF), overlap = 3cm, suture: Ethibond 3-0, porcine tendons

	Test No.	Date	Porcine limb	Date of Preparation	ture Techniq	ndon combinati	Length of Overlap in mm	iameter of Donortendon in m	ameter of Recipient Tendon in m	Diameter Suture small in mm	Diameter Suture big in mm	ross Sectional Area 2 Tendons in mm	s Sectional Area Suture in	train during First Failure in mn	FFL First Failure Load in N	in during Ultimate Load in	ax Ultimate Load i	S Stiffness in N/mm	Tendon Failure
**Pulvertaft**	1	21.02.19	Pfote 3	13.02.19	PT	lat/dist	23,8	2,4	2,7	3,4	3,8	10,2	10,1	11,89	141,00	11,89	141,00	19,95	D&R
	7	21.02.19	Pfote 4	13.02.19	PT	med/dist	23,8	2,4	3,1	4,6	5,2	12,1	18,8	10,41	117,08	14,77	135,36	18,05	D
	2	22.02.19	Pfote 1	15.02.19	PT	lat/prox	24,1	2,2	3,7	5,0	6,0	14,6	23,6	12,69	167,49	12,69	167,49	23,46	D
	6	22.02.19	Pfote 2	15.02.19	PT	lat/dist	24,1	2,1	2,4	3,9	4,6	8,0	14,1	7,86	87,62	9,93	88,83	18,70	D
	9	22.02.19	Pfote 2	15.02.19	PT	med/prox	24,2	1,8	2,7	4,0	5,3	8,3	16,7	8,33	59,52	9,23	59,72	16,22	D
	2	25.02.19	Pfote 3	15.02.19	PT	med/dist	23,2	1,9	3,5	4,6	6,3	12,5	22,8	11,46	127,04	11,46	127,04	20,97	D
	5	25.02.19	Pfote 4	15.02.19	PT	lat/prox	23,0	2,4	3,2	4,0	6,3	12,6	19,8	8,94	87,69	8,94	87,69	18,81	D&R
	1	27.02.19	Pfote 1	26.02.19	PT	lat/dist	24,3	2,8	2,2	3,6	5,1	10,0	14,4	9,84	138,51	9,84	138,51	23,13	R
	4	27.02.19	Pfote 1	26.02.19	PT	med/prox	25,2	2,1	3,2	4,3	5,8	11,5	19,6	8,79	113,36	11,59	121,74	19,83	D
	7	27.02.19	Pfote 2	26.02.19	PT	med/dist	27,4	2,2	3,3	4,2	5,7	12,4	18,8	15,27	141,12	15,27	141,12	15,64	D
	10	27.02.19	Pfote 3	26.02.19	PT	lat/prox	24,3	2,5	3,3	4,8	5,0	13,5	18,8	15,62	192,14	15,62	192,14	21,20	D
	13	27.02.19	Pfote 4	26.02.19	PT	med/prox	22,8	2,3	3,0	4,3	5,1	11,2	17,2	12,22	84,02	12,22	84,02	14,76	D
**Fridén**	2	21.02.19	Pfote 3	13.02.19	FR	lat/prox	25,5	2,0	3,1	2,2	6,1	10,7	10,5	8,51	123,12	10,17	127,04	23,09	D
	5	21.02.19	Pfote 4	13.02.19	SA	lat/dist	26,2	1,8	2,2	2,3	5,5	6,3	9,9	11,81	125,64	16,17	131,87	16,84	D&R
	8	21.02.19	Pfote 4	13.02.19	SO	med/prox	27,9	2,3	3,0	4,1	5,0	11,2	16,1	12,04	152,14	16,17	157,41	17,19	D
	3	22.02.19	Pfote 1	15.02.19	MO	med/dist	27,0	2,1	4,1	4,2	6,2	16,7	20,5	8,79	141,71	12,73	156,67	26,65	D
	7	22.02.19	Pfote 2	15.02.19	DI	lat/prox	25,2	2,5	3,1	3,0	5,1	12,5	12,0	9,48	178,21	9,48	178,21	31,40	D&R
	3	25.02.19	Pfote 3	15.02.19	MI	med/prox	26,8	1,9	3,0	4,0	5,7	9,9	17,9	10,81	136,85	10,81	136,85	20,39	D
	6	25.02.19	Pfote 4	15.02.19	DO	med/dist	26,2	2,6	3,4	3,4	5,8	14,4	15,5	8,63	163,22	8,63	163,22	31,09	D
	2	27.02.19	Pfote 1	26.02.19	FR	lat/prox	26,5	2,5	2,7	3,3	5,6	10,6	14,5	10,16	175,36	10,16	175,36	27,16	D
	5	27.02.19	Pfote 2	26.02.19	SA	lat/dist	26,1	2,4	2,4	2,9	5,3	9,0	12,1	10,06	146,63	10,06	146,63	24,04	D
	8	27.02.19	Pfote 2	26.02.19	SO	med/prox	27,8	2,1	2,9	4,0	5,5	10,1	17,3	11,07	170,31	11,07	170,31	24,09	D
	11	27.02.19	Pfote 3	26.02.19	MO	med/dist	27,6	2,1	3,6	3,8	6,1	13,6	18,2	11,79	221,48	13,39	222,70	34,43	D
	14	27.02.19	Pfote 4	26.02.19	DI	lat/dist	25,1	1,9	2,0	2,5	4,5	6,0	8,8	8,61	120,05	8,61	120,05	23,51	D&R
**Woven Fridén**	3	21.02.19	Pfote 3	13.02.19	WF	med/dist	26,5	2,1	3,3	2,7	6,8	12,0	14,4	14,34	174,07	14,34	174,07	18,90	D
	6	21.02.19	Pfote 4	13.02.19	WF	lat/prox	25,4	2,5	2,5	2,7	5,0	9,8	10,6	11,02	217,02	11,02	217,02	29,87	D
	1	22.02.19	Pfote 1	15.02.19	WF	lat/dist	26,2	2,3	3,6	3,0	5,6	14,3	13,2	10,78	177,25	10,78	177,25	26,57	D
	4	22.02.19	Pfote 1	15.02.19	WF	med/prox	27,3	2,1	3,2	3,8	5,7	11,5	17,0	10,78	201,05	10,78	201,05	29,54	D
	8	22.02.19	Pfote 2	15.02.19	WF	med/dist	26,8	2,2	3,3	2,7	6,9	12,4	14,6	9,86	171,20	9,86	171,20	27,28	D
	1	25.02.19	Pfote 3	15.02.19	WF	lat/prox	24,6	2,2	2,9	2,5	6,4	10,4	12,6	10,28	208,84	10,28	208,84	31,15	D
	4	25.02.19	Pfote 4	15.02.19	WF	lat/dist	24,0	2,6	2,4	2,8	5,7	9,8	12,5	11,94	152,35	11,94	152,35	21,41	R
	7	25.02.19	Pfote 4	15.02.19	WF	med/prox	26,9	1,7	3,3	2,4	5,4	10,8	10,2	8,74	149,18	8,74	149,18	28,91	D
	3	27.02.19	Pfote 1	26,02,19	WF	med/dist	26,8	2,1	3,6	2,2	6,8	13,6	11,7	9,84	183,20	9,84	183,20	29,69	D
	6	27.02.19	Pfote 2	26,02,19	WF	lat/prox	25,1	2,6	3,9	2,9	6,1	17,3	13,9	12,14	209,05	12,14	209,05	27,53	D
	9	27.02.19	Pfote 3	26,02,19	WF	lat/dist	26,0	2,6	2,7	2,5	5,9	11,0	11,6	12,66	244,51	12,66	244,51	28,96	D
	12	27.02.19	Pfote 3	26,02,19	WF	med/prox	27,3	2,6	3,0	3,6	6,7	12,4	18,9	13,07	241,60	13,07	241,60	27,79	D

	**Mean Fmax in N**	**Mean FFL in N**	**Mean S in N/mm**
**Pulvertaft**	123,72	121,38	19,23
**Fridén**	157,19	154,56	24,99
**Woven Fridén**	194,11	194,11	27,30
	**Standard Deviaton Fmax in N**	**ndard Deviation FFL in N**	**Standard Deviation S in N/mm**
**Pulvertaft**	37,85	37,92	2,78
**Fridén**	28,17	29,17	5,48
**Woven Fridén**	31,48	31,48	3,61

	Mean Length of Overlap	mean Cross Sectional Area n	ss Sectional Area Suture in mm2	Bulk ratio	Mean Diameter Donor Tendon in	Mean Diameter Recipient Tendon in mm
Pulvertaft	24,2	11,4	17,9	158%	2,3	3,0
Fridén	26,5	10,9	14,4	136%	2,2	3,0
Woven Fridén	26,1	12,1	13,4	113%	2,3	3,1
	Standard Deviation Lengt	Standard Deviation Cross Sec	ss Sectional Area Suture in mm2	Standard Deviation Bulk Ratio	0,3	0,4
Pulvertaft	1,2	2,0	3,7	25%	0,3	0,6
Fridén	1,0	3,1	3,7	27%	1,1	0,5
Woven Fridén	1,1	2,1	2,6	23%		

	Donor Failure	Donor and Recipient Failu	Recipient Failure
Pulvertaft	75%	17%	8%
Fridén	75%	25%	0%
Woven-Fridén	92%	0%	8%

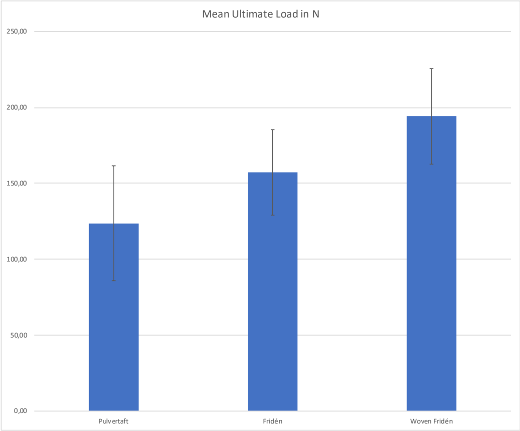

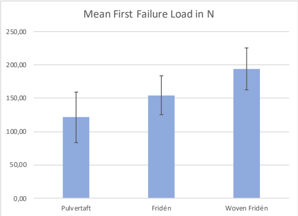

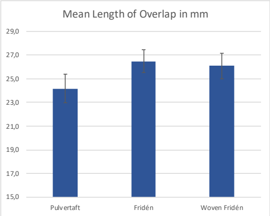

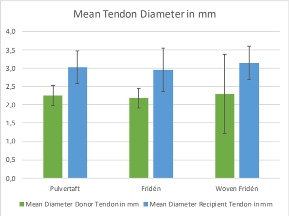

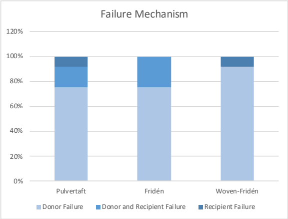

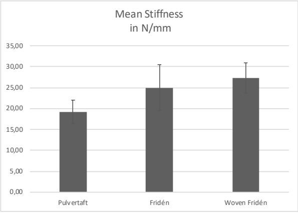

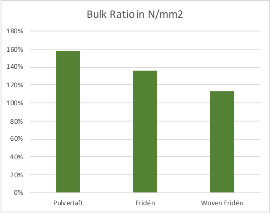

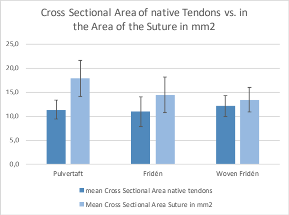

Supplement: S1 Data — (PDF) [file pone.0257038.s004.pdf]
